# Colon capsule endoscopy leading to gastrointestinal stromal tumor (GIST) diagnosis after colonoscopy failure

**DOI:** 10.1186/s13104-015-1444-x

**Published:** 2015-10-12

**Authors:** A. Stemate, A. M. Filimon, M. Tomescu, L. Negreanu

**Affiliations:** Internal Medicine 2-Gastroenterology Department, University Hospital, Carol Davila University of Medicine Bucharest, 169, Splaiul Independentei Street, Sector 5, Bucharest, Romania; Vascular Surgery Department, University Hospital, Carol Davila University of Medicine Bucharest, V Babes National Institute of Pathology, Bucharest, Romania; General Surgery Department, University Hospital, Carol Davila University of Medicine Bucharest, Bucharest, Romania

**Keywords:** Gastrointestinal stromal tumors, GIST, Sigmoid colon, Colonoscopy failure, Capsule endoscopy, Pillcam colon2

## Abstract

**Background:**

Gastrointestinal stromal tumors are a subtype of mesenchymal tumors. In recent years a significant progress was made in their diagnosis and treatment which led to significant improvement of their prognosis. Endoscopy remains one of the main diagnostic methods. In the rare instance of colonoscopy failure, different approaches are available: different endoscope, computed tomography colonography, capsule endoscopy, use of an enteroscope.

**Case presentation:**

We present the case of a 75-year-old Caucasian man admitted for abdominal pain, diarrhea and weight loss. Two colonoscopy attempts failed in a different center and a decision to use colon capsule endoscopy was made. This exam revealed a submucosal mass located in the sigmoid colon. Surgery was performed and a local invading gastrointestinal stromal tumor was removed. This is the first image of a colonic gastrointestinal stromal tumor seen on capsule endoscopy.

**Conclusion:**

Colon capsule is a useful diagnostic tool in selected patients after colonoscopy failure or contraindication.

## Background

Gastrointestinal stromal tumors are a subtype of mesenchymal tumors. Even if they represent only 1 % of the digestive tumors, they had reached celebrity in the last years, due to better understanding of their pathogenesis and progress in their diagnosis due to the new and improved imaging tools. Also, a significant progress was made with a targeted molecular treatment which led to significant improvement of their prognosis.

The most common locations of GIST’s are the stomach (50–60 %) and small intestine (30–40 %) and only less then 5–10 % develops in colon, rectum and 1 % in esophagus [1, 2]. Similar tumors may arise in omentum, mesentery, retroperitoneum, pancreas, liver, gallbladder and urinary bladder. These tumors are called extragastrointestinal stromal tumors (EGISTs) [3, 4].

Endoscopy, echoendoscopy and computed tomography scan are the mean diagnostic methods. We present the case of a patient with a sigmoid colon located GIST, where diagnosis was established using capsule endoscopy after a failed colonoscopy examination.

## Case presentation

A 75-year-old Caucasian man presented to our department with recurrent left lower quadrant abdominal pain, weight loss and progressive asthenia. The stools were occasional diarrheic, with no visible blood. The symptoms started a few months earlier and they gradually increased. He had no significant medical history.

The clinical examination showed a pale patient, with left lower abdominal quadrant pain, no palpable masses, no ascites and no peripheral edema. The blood tests were within normal limits except mild iron deficiency anemia (hemoglobin 10.2 g/dl, serum iron 33 μg/dl) and an inflammatory syndrome (positive C—reactive protein, high erythrocyte sedimentation rate 42 mm/h).

The patient had been admitted in a different department in the previous month, where an upper endoscopy that found no lesions and two colonoscopies attempts which failed to pass the sigmoid colon were performed. He underwent a barium enema and he was diagnosed with diverticulosis. His symptoms were interpreted as a diverticulitis attack. He received anti-diarrheics, spasmolytics, and a fluoroquinolone with partial and temporary symptom alleviation.

He was referred to our unit for a second opinion. Since he had two failures of colonoscopy and since we had an ongoing study using the Pillcam Colon2 videocapsule for the patients unable or unwilling to undergo colonoscopy, we decided to use the Pillcam Colon2 videocapsule from Given Imaging for the colonic exploration. The use of colon capsule endoscopy was not standard care at our institute at that time, but we considered it was the best alternative for our patient. Lately, according to the current European Society of Gastrointestinal Endoscopy capsule endoscopy guidelines, CCE is feasible and safe for visualization of colonic mucosa in patients with incomplete colonoscopy and without stenosis. [8].

**Fig. 1:**
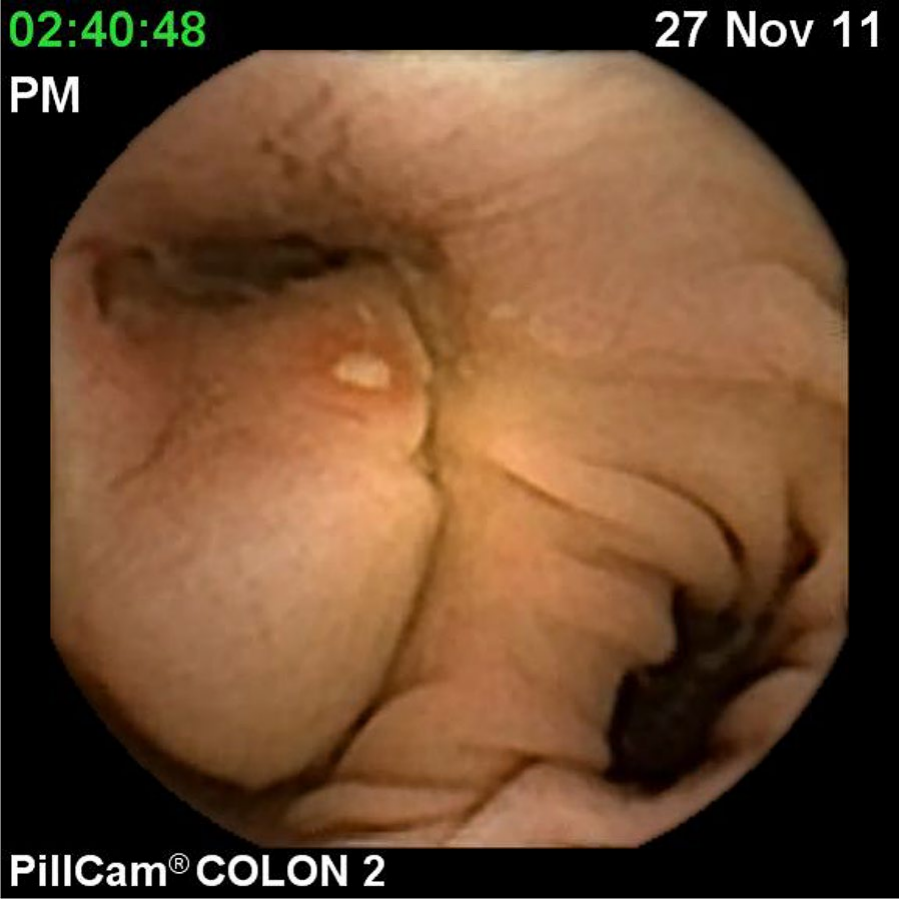
Ulcerated submucosal mass in the sigmoid colon

After review of his barium irigography, no colonic stenosis was suspected. A complete colonic examination was obtained by capsule and showed multiple diverticula with no signs of inflammation of the surrounding mucosa. However, we noticed a submucosal mass approximately 3 cm in diameter located in the sigmoid colon Fig. 1.

A CT scan of the abdomen and the pelvis showed multiple colonic diverticula and a soft tissue mass of 5 cm diameter with a necrotic centre, with increased thickness of the sigmoid wall. No clear demarcation between the mass and the urinary bladder was visible and important densification of the perisigmoidian fat was also noticed. Although a peridiverticular abscess was possible, viewing the CT aspect corroborated with the capsule images, the diagnosis of a tumor with necrotizing center, probably a GIST it was more likely, as this kind of necrosis is quite specific and previously described in GIST’s [5]. The patient underwent surgery with complete tumor resection. The lymph nodes examined during surgery were not involved.

The histopathological examination report revealed a sigmoid GIST with a tumor size more than 5 cm, with spindle cells, CD117 positive and a mitotic rate more than 5/50 HPF and since the patient had no significant comorbidities we considered him for the adjuvant therapy. The patient started treatment with imatinib mesylate, with good evolution at 6 months.

## Discussion

The clinical features of GIST’s are variable, related to the presence of mass, perforation or obstruction. The presentation can be with abdominal pain or discomfort, acute or chronic bleeding or anorexia [1]. Some patients remain asymptomatic and some GISTs are detected by random imagistic examinations or at autopsy.

Specific morphological and immunohistochemical features establish the definite diagnosis of GIST: they are formed by spindle or epithelioid cells which are expressing CD117 and mutations in KIT or PDGFRα (platelet derived growth factor alpha). Approximately 80 % also express CD34. Less then 5 % are c-KIT negative [6].

Endoscopy is one of the main methods of diagnosis. Although the rate of colonoscopy completeness is as high as 97 % in expert centers, a variable proportion (4–20 %) of patients will have an incomplete colonoscopy [7].

After an incomplete colonoscopic examination different approaches can be tried: variable stiffness colonoscope, use of gastroscope, single or double balloon enteroscopy (not readily available in all centers). Changing the centre or the endoscopist is an alternative. However, a first failed colonoscopy is more commonly associated with a lower future success rate, particularly when stopped in the sigmoid [7]. Radiological methods as double contrast barium enema or CT colonography are also useful examination tools. Lately, colon capsule endoscopy was regarded as a possible alternative for such patients. The current European Society of Gastrointestinal Endoscopy capsule endoscopy guideline takes into consideration the utilization of CCE after failure or refuse of colonoscopy. According to these guidelines, CCE is feasible and safe and appears to be accurate when used in average-risk individuals and in high risk patients for whom colonoscopy is inappropriate or not possible. For these patients the use of CCE could be an alternative [8].

At the moment four studies address this issue, three using the first generation colon capsule, the other, from our center, presenting the results of the second generation colon capsule. All studies conclude that colon capsule endoscopy can be very useful in the setting of selected patients after colonoscopy failure or contraindication [9–12]. In this particular case the colon capsule permitted the visualization of a mass in sigmoid (not seen in barium contrast enema) and played a crucial role in the differential diagnosis with diverticulitis. All GIST’s have a malignant potential with metastases being detected at the moment of diagnosis in 50 % of cases. The common metastatic sites of GIST’s are the liver and the peritoneum with rare lymph node involvement occurring in only 0–8 % of cases. The prognosis depends on the site and size of the tumor and mitotic rate. Small tumors (<2 cm) and mitotic activity of less then 5 mitoses per 50 HPFs have an excellent prognosis, probably independent of site. The Ki67 index may help identify tumors with malignant potential [13].

Complete surgical resection is the treatment of choice for GIST’s [14]. Imatinib mesylate is a synthetic tyrosine kinase inhibitor and is considered the drug of choice for metastatic and inoperable GISTs [15]. Also, from 2008, the FDA approved imatinib mesylate for the adjuvant therapy of primary resected GIST [16].

## Conclusion

The colonic GIST’s are rare and a proper evaluation, diagnosis and treatment are needed for the best outcome. In the instance of failing colonoscopy, a useful alternative is colon capsule endoscopy.

This is the first case to our knowledge, presenting the capsule image of a colonic GIST. In our patient the capsule endoscopy made the difference, making the correct diagnosis possible and permitting a specific treatment.

## Abbreviations

GIST: gastrointestinal stromal tumor
EGISTs: extragastrointestinal stromal tumors
CT: computed tomography
HPF: high-power field
PDGFR: platelet derived growth factor receptor
CCE: colon capsule endoscopy
FDA: Food and Drug Administration
